# Modeling a 3-D multiscale blood-flow and heat-transfer framework for realistic vascular systems

**DOI:** 10.1038/s41598-022-18831-3

**Published:** 2022-08-26

**Authors:** Rohan Amare, Erlend Hodneland, Jeremy A. Roberts, Amir A. Bahadori, Steven Eckels

**Affiliations:** 1 Institute for Environmental Research, Kansas State University, Manhattan, KS USA; 2 Alan Levin Department of Mechanical and Nuclear Engineering, Kansas State University, Manhattan, KS USA; 3 Department of Mathematics, University of Bergen, Bergen, Norway; 4 MMIV Mohn Medical Imaging and Visualization Centre, Haukeland University Hospital, Bergen, Norway; 5 Radiological Engineering Analysis Laboratory, Kansas State University, Manhattan, KS USA

**Keywords:** Computational models, Biomedical engineering, Computational biophysics

## Abstract

Modeling of biological domains and simulation of biophysical processes occurring in them can help inform medical procedures. However, when considering complex domains such as large regions of the human body, the complexities of blood vessel branching and variation of blood vessel dimensions present a major modeling challenge. Here, we present a Voxelized Multi-Physics Simulation (VoM-PhyS) framework to simulate coupled heat transfer and fluid flow using a multi-scale voxel mesh on a biological domain obtained. In this framework, flow in larger blood vessels is modeled using the Hagen–Poiseuille equation for a one-dimensional flow coupled with a three-dimensional two-compartment porous media model for capillary circulation in tissue. The Dirac distribution function is used as Sphere of Influence (SoI) parameter to couple the one-dimensional and three-dimensional flow. This blood flow system is coupled with a heat transfer solver to provide a complete thermo-physiological simulation. The framework is demonstrated on a frog tongue and further analysis is conducted to study the effect of convective heat exchange between blood vessels and tissue, and the effect of SoI on simulation results.

## Introduction

Technological advancements are enabling visualization and modeling of the vasculature^1,2^ with ever-increasing resolution, providing highly detailed blood vessel domains. These domains can be further used to simulate many bio-physical mechanisms. Such realistic models, when coupled with accurate simulation of bio-physics, can be used to illustrate, understand, and predict biological response to different environmental conditions. Such a tool can be used for predicting patient response to a medical treatment, changes in blood flow distribution due to burns or clots^3–5^, drug distribution, and damage to healthy tissue during hyperthermia treatments^6,7^.

There are three major challenges involved in creating such a realistic simulation model. The first challenge is to generate a biological domain for simulation that can be made to represent individual patients with relative ease^8,9^. The most straightforward method to achieve this is to use imaging data (e.g., computed tomography and magnetic resonance imaging) to generate voxelized domains. Varieties of voxel phantoms^6,10–13^ are used in biomedical applications^10^ and radiation dosimetry studies^14–16^.

The second challenge associated with such voxelized domains is limitations associated with image resolution. Achieving a complete and continuous blood vessel network is difficult when modeling voxelized phantoms. Blood vessel radii span the micrometer to centimeter scales, with a 1.25 cm radius at the aorta, 3 $\upmu$m at the capillary bed, and 1.5 cm at vena cava^17^. Existing in vivo imaging technology does not allow this fine resolution of microns. Clinical scanners typically provide images with voxel dimensions in the range of millimeters^18^. This resolution cannot represent the finer blood vessel branches, and so such anatomical structures are absent in voxel phantoms, resulting in an incomplete and discontinuous blood vessel network. To model the capillary bed and blood flow in such a domain, porous media methods are typically employed^19–23^.

The third challenge is to accurately simulate the effect of blood flow on heat transfer. Several approaches have been used to model such coupled multi-physics phenomena^21,22,24–27^. The assumptions associated with each of these models can differ substantially. For the Pennes Bioheat Model (PBM)^24^, it is assumed that blood does not exchange heat with tissue via convection and achieves immediate thermal equilibrium with tissue upon delivery. In the Weinbaum and Jiji Model (WJM)^26^, incomplete counter-current heat exchange in thermally significant micro-vessels is assumed to be the primary mechanism for blood-tissue heat exchange. While the PBM gives more importance to heat transfer occurring in capillary bed, the WJM gives more importance to heat transfer resulting from counter-current heat exchange between arteries, veins, and tissue. Complexities associated with the blood vessel network and the lack of experimental data to validate bioheat transfer simulations leads to such extreme variations in model assumptions.

These three challenges are addressed through the use of mixed-dimensional models (e.g.^28–30^). Of these, the VaPor model proposed by Blowers et al.^28^ is used for thermal analysis^31^. The VaPor model employs the Rapidly-exploring Random Tree (RRT) algorithm^32^ to generate blood vessels that are not segmented due to limitations in image resolution and simulates counter-current heat exchange at every level of the vasculature. At the end of each vessel terminal, only the voxels that intersect with the vessel exchange blood. Other voxels that do not intersect with any terminal vessel rely on perfusion for blood flow. The inter-domain mass transfer thus becomes an important parameter, which is specified at each vessel terminal. This parameter is further used to determine the diameters of blood vessels and to calculate pressure drop across the vessel segments. In human thermal modeling research^10,27,33–35^, the pressure drop across the cardiovascular domain is an important parameter. The blood pressure and resultant blood circulation affects thermal response of body. Similarly, hyperthermia affects the cardiac response^36^. The VaPor model provides a novel method to simulate heat transfer in a mixed domain but lacks the ability to couple pressure gradient with assigned inter-domain mass transfer^28^. Furthermore, determining the flow rate at every vessel terminal is challenging when simulating a very large domain, such as the human body, especially when vasomotion is an important aspect of thermoregulation and controls the blood distribution.

Here, we present a Voxelized Multi-Physics Simulation (VoM-PhyS) framework to model and simulate a complex biological domain using realistic vasculature obtained from imaging data. This framework employs the flow simulation method proposed by Hodneland et al.^37^, which uses the Hagen–Poiseuille equation to model one-dimensional (1D) blood flow in large blood vessels coupled with a three-dimensional (3D) porous media model to simulate the capillary bed in tissue that cannot be segmented due to limitations in image spatial resolution. In contrast with the VaPor model, 1D blood vessel and 3D porous voxel coupling is achieved using a Dirac distribution function.

The flow simulation then coupled with a heat transfer model, completes the VoM-PhyS framework that addresses the three previously described challenges. The framework is further used to analyse the effect of convective heat transfer between blood vessels and tissue, and the effect of the Dirac distribution method.

## Methods

Macro- and micro-scale blood flows are modeled in coupled fashion for continuous blood flow. The macro-scale blood flow model employs the Hagen–Poiseuille equation in a 1D flow domain. The 3D micro-scale blood flow is modeled with the two-compartment model theory^5,38^ and the Darcy equation for porous media.

**Figure 1:**
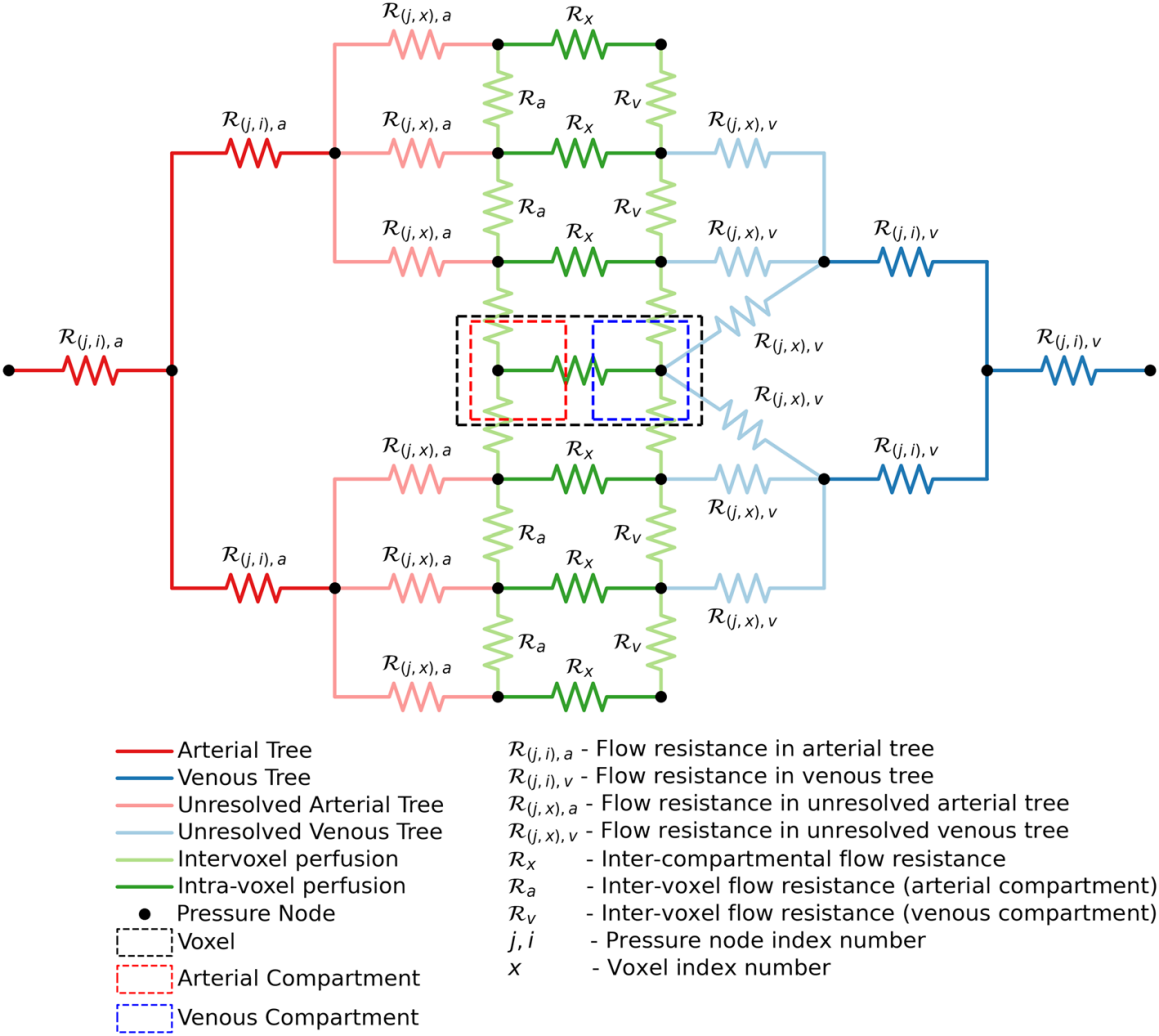
Proposed resistance model of Hodneland et al.^37^. The arterial tree and venous tree is modeled as 1D pipe flow network. The resistance offered by each of these tree elements is calculated using the Hagen–Poiseuille equation (Eq. (1)). Each voxel has two compartments—arterial and venous to represent tissue, capillary bed, and capillary perfusion. The blood perfusion in tissue voxels is 3D. The inter-voxel compartmental flow is modeled using Darcy’s equation (Eq. (2)) and estimation of tissue permeability obtained from the literature. Blood perfusion from arterial compartment to venous compartment in a voxel is calculated using Eq. (3). The unresolved arterial and venous tree represents the Dirac distribution function (Eq. (6)) used to couple 1D flow in resolved network with 3D perfusion in tissue voxels.

### Blood flow modeling

An illustration of the blood flow framework of Hodneland et al.^37^ using resistances is shown in Fig. 1. Blood vessels that can be recreated from imaging data are represented in red for arteries and blue for veins in the figure. The flow resistance offered by each element of these blood vessels is represented by ${\mathcal{R}}_{ji}$. Each element has two nodes for flow inlet and outlet, with element nodal numbers represented by ‘$\text{j}$’ and ‘$\text{i}$’ subscripts, respectively. These vessels are modeled in 1D using the Hagen–Poiseuille equation (Eq. (1)).

1 $$\begin{aligned} \text{q}_\text{ji} = {\kappa _\text{ji}}{\Delta }\text{p}_\text{ji}, \end{aligned}$$

where

$$  \begin{gathered}   {\mathcal{R}}_{ji}^{ - 1}  = \kappa _{{{\text{ji}}}}  = \frac{{\pi {\text{R}}_{{{\text{ji}}}}^{4} }}{{8\mu {\text{L}}_{{{\text{ji}}}} }}, \hfill \\   \Delta {\text{p}}_{{{\text{ji}}}}  = {\text{p}}_{{\text{j}}}  - {\text{p}}_{{\text{i}}}  \hfill \\  \end{gathered}  $$

In Eq. (1), the flow conductivity offered by a blood vessel element is represented as $\kappa_ \text{{ji}}$, the net flow from an element is given by $\text{q}_\text{ji}$, and the radius and length of the specific element are represented by $\text{R}$ and $\text{L}$, respectively. The nodes represented by ‘$\text{j}$’ and ‘$\text{i}$’ are the locations for which pressure is calculated and are represented as black dots (pressure nodes) in Fig. 1. The pink and light blue resistances in Fig. 1 represent blood vessels that cannot be recreated from imaging data due to spatial resolution limitations. The pressure drop parameter (${\gamma _{\beta }}$) is used to calculate effective resistance (${\mathcal{R}}_{(j,x),\beta }$) in the unresolved network extending from the resolvable blood vessels to tissue voxels^37^.

The 3D voxel domain consists of tissue and a capillary bed. Each voxel has two compartments: one representing the arterial capillary bed and tissue (referred to as arterial compartment), and the other representing the venous capillary bed and tissue (referred to as venous compartment). Darcy’s equation (Eq. (2)) provides the relation between mass flux *u* and pressure drop across the porous domain. The viscosity $\mu$ is considered constant and *k* represents the permeability of the porous domain. In the current study, *k* is the vascular permeability in tissue voxel. The cross-voxel flow resistance (${\mathcal{R}}_{a}, {\mathcal{R}}_v$), shown using the light green resistance network in Fig. 1, controls the distribution of blood across neighboring voxels. The subscripts ‘$\text{a}$’ and ‘$\text{v}$’ denote the arterial and venous compartment properties, respectively. The estimation of tissue permeability (${k_{a}}$, ${k_{v}}$) is calculated as given in Ref.^39^

2 $$\begin{aligned} u = -\frac{k}{\mu } \nabla P. \end{aligned}$$

Perfusion between the arterial compartment and the venous compartment represents the transition of blood from oxygenated to deoxygenated state. This perfusion is driven by pressure difference and perfusion proportionality factor $\alpha$ as shown in Eq. (3)

3 $$\begin{aligned} u_{perf} = \alpha (P_a - P_v). \end{aligned}$$

The dark green resistance shown in Fig. 1 represents perfusion resistance ${\mathcal{R}}_x$ from the arterial compartment to the venous compartment within the same voxel ‘*x*’. The perfusion parameter $\alpha$ controls the resistance offered to the flow between the compartments in a voxel of volume $\text{V}$.

The mass conservation equation for an incompressible fluid at steady state and constant density (Eq. (4)), when applied to a porous domain with two compartments, results in Eq. (5)

4 $$\begin{aligned}&\nabla \cdot u = {\mathcal{Q}}, \end{aligned}$$

5 $$\begin{aligned}&-\nabla \cdot \left( \frac{k_a}{\mu } \nabla P_a \right) = -\alpha (P_a - P_v) + \sum _{i \in N_a^T}{\mathcal{Q}}_{a,i}, \nonumber \\&-\nabla \cdot \left( \frac{k_v}{\mu } \nabla P_v \right) = \alpha (P_a - P_v) - \sum _{i \in N_v^T}{\mathcal{Q}}_{v,i}. \end{aligned}$$

Equation (5) represents the mass conservation equation for the arterial and venous compartments. In an arterial compartment, the oxygenated blood enters from the arteries and is represented as a mass source term ${\mathcal{Q}}_a$. Similarly, in the venous compartment, the deoxygenated blood leaves the tissue and enters the veins. This is represented by a sink term ${\mathcal{Q}}_v$ in the venous compartment. The perfusion that carries blood from arterial to venous compartment acts as sink and source term, respectively. ${\mathcal{Q}}_{a,i}$ represents the mass flow rate of blood arriving at the arterial compartment from an ‘i’-th arterial terminal. A voxel can receive blood from multiple arterial terminals ($N_a^T$). Similarly, ${\mathcal{Q}}_{v,i}$ represents the mass flow rate of blood leaving the tissue to ‘i’-th venous vessels. A voxel can exchange blood with multiple venous terminals ($N_v^T$). The amount of blood flow exchange (${\mathcal{Q}}_{a,i}$, ${\mathcal{Q}}_{v,i}$) that occurs between a tissue voxel and a blood vessel is determined using a Dirac function (Eq. (6)).

6 $$\begin{aligned} Q^{\epsilon }(x) = \int _{\Omega } Q(y) \eta ^{\epsilon } (x-y)dy, \end{aligned}$$

where

6a $$\begin{aligned} \eta ^{\epsilon } (x) = \frac{1}{\epsilon ^n} \eta \bigg (\frac{x}{\epsilon }\bigg ), \end{aligned}$$

6b $$\begin{aligned} \eta (x) = {\left\{ \begin{array}{ll} C \exp \left( \frac{1}{|x|^2 - 1}\right) , &{} \text{if } |x| < 1 \\ 0, &{} \text{if } |x| \ge 1 \end{array}\right. }, \end{aligned}$$

6c $$\begin{aligned} \int _{\Omega } \eta ^{\epsilon } (x)dx = 1. \end{aligned}$$

Equation (6) is a distribution function of flow between the terminal points of the arterial or venous tree and the voxels in the neighborhood of the terminal. The distribution function is applied over the computational domain $\Omega$. *Q*(*y*) represents the flow in the terminal arterial and venous elements. Considering an arterial tree, *Q*(*y*) represents flow in the terminal arterial element, and a voxel at location *x* receives $Q^{\epsilon } (x)$ amount of flow from the respective terminal arterial element. The amount of flow that a voxel receives from a specific arterial element is controlled by how far the voxel is from the artery, given by $\eta ^ {\epsilon } (x-y)$. The distribution given by the function $\eta ^{\epsilon } (x)$ depends on the characteristic radius $\epsilon$ and constant *C*. The superscript *n* in Eq. (6a) is the number of dimensions of simulation domain. The characteristic radius is the radius of the sphere of influence (SoI) which exchanges blood with the terminal blood vessel elements. The value of constant *C* depends on characteristic radii and is calculated using Eq. (6c). Equation (6c) conserves the mass in the virtual unresolved blood vessels. The final finite volume two-point flux approximation (TPFA)^40^ provides the discretized flow equation for the voxel domain given in Eqs. (7) and (8) for the arterial and venous compartments, respectively^37^.

7 $$\begin{aligned}&\sum _{j \in N} t_{ij} (P_{a,i} - P_{a,j}) + \alpha _i (P_{a,i} P_{v,i}) \text{V}_i \nonumber \\&\quad - \sum _{k \in N_{a}^{T} } q_{a,k} \eta _{a,k} ^{\epsilon } (x_i-x_k) \text{V}_i = 0, \end{aligned}$$

where

$$\begin{aligned} q_{a,k} = \kappa _{a,jk} (p_{a,j} - p_{a,k}) \quad \quad j \in {\mathcal{N}}\left( N_{a,k}^T\right) , \end{aligned}$$

and

8 $$\begin{aligned}&\sum _{j \in N} t_{ij} (P_{v,i} - P_{v,j}) - \alpha _i (P_{a,i} - P_{v,i}) \text{V}_i - \nonumber \\&\quad - \sum _{k \in N_{v}^{T}} q_{v,k} \eta _{v,k} ^{\epsilon } (x_i-x_k) \text{V}_i = 0, \end{aligned}$$

where

$$\begin{aligned} q_{v,k} = \kappa _{v,jk} (p_{v,j} - p_{v,k}) \quad \quad j \in {\mathcal{N}}\left( N_{v,k}^T\right) , \end{aligned}$$

In Eqs. (7) and (8) *N* represents the number of neighboring voxels exchanging blood with voxel ‘*i*’ and, $N_a^T$ and $N_v^T$ represent the terminal arterial and venous nodes exchanging blood with voxels. ${\mathcal{N}}$ represents the nodes that are neighboring the ‘k’-th blood vessel element. The sub-script ‘*j*’ represents the node of a terminal blood vessel element which is connected to node ‘*k*’. The pressure drop across these two nodes results in flow rates across the terminal blood vessels. The last equation to complete the system is the pressure continuity equation (Eq. (9)),

9 $$\begin{aligned} q_{\beta ,k}=\frac{\gamma _{\beta }}{\mu }(p_{\beta ,k} - \sum _{j \in N_{\beta ,k}^V} \bigg (\eta _{\beta ,k}^{\epsilon }(x_j - x_k)P_{\beta ,j} \text{V}_j\bigg ) \quad \quad k \in N_{\beta }^T, \end{aligned}$$

$$\begin{aligned} {\beta } = {\left\{ \begin{array}{ll}\text{a: artery} \\ \text{v: vein} \end{array}\right. }. \end{aligned}$$

Equation (9) represents the pressure continuity across the virtual blood vessels which are modeled using Eq. (6). $N_{\beta }^T$ represents all the terminal blood vessels and $N_{\beta ,k}^{V}$ represents the set of tissue voxels that fall within the SoI of vessel terminal $`k'$.

Equations (1), (7), (8) and (9) provide a set of equations that can be solved to calculate pressure at each pressure node when applied to blood vessel elements and arterial and venous compartments of voxels. A more detailed description and derivation of these equations can be found in Refs.^37,40,41^. A detailed description of matrix generation using these equations is presented in Supplementary Appendix A1.

**Figure 2:**
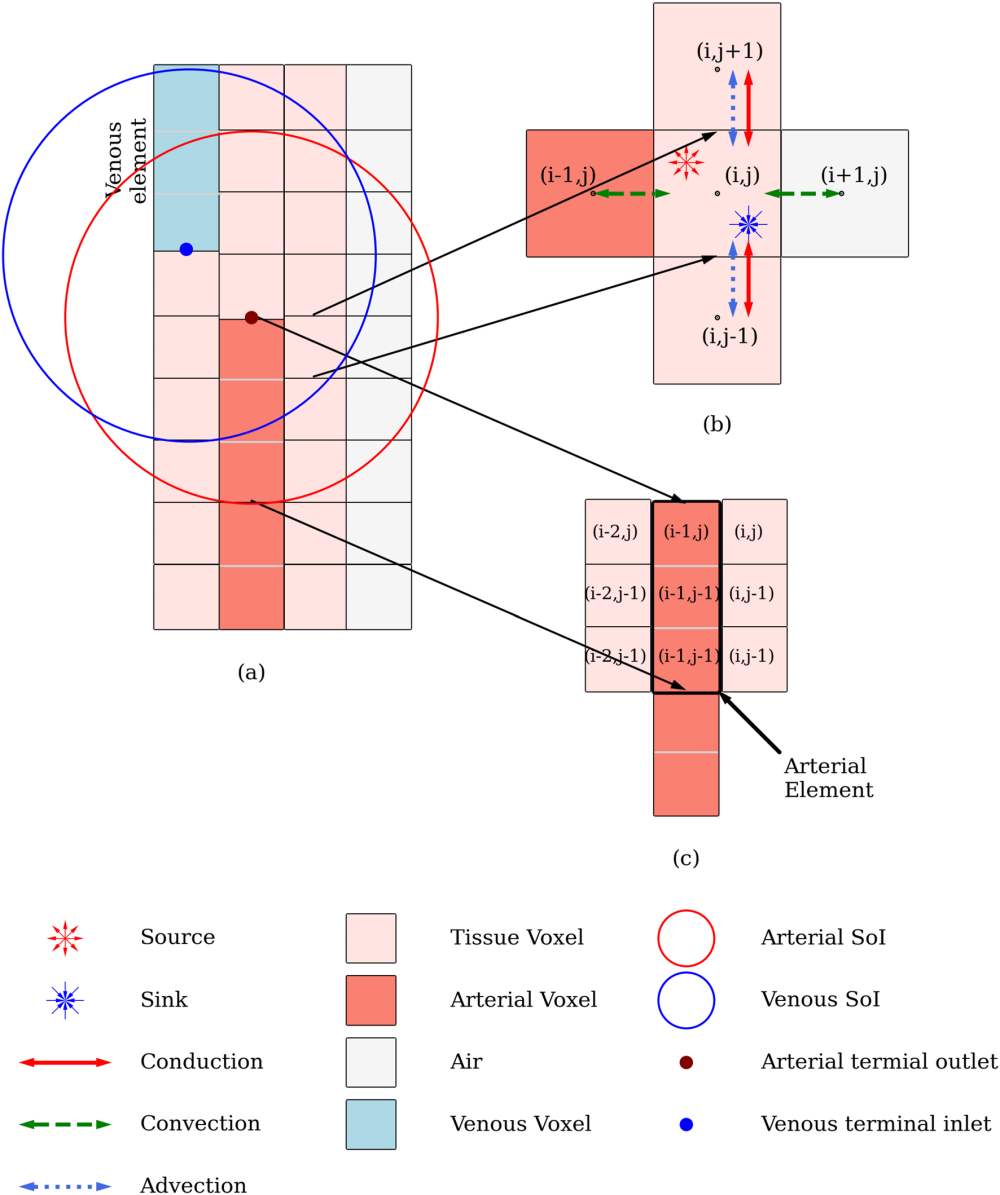
An illustrative description of the Heat transfer model. (**a**) A small voxel domain representing artery, vein, and tissue, with SoI for an artery and vein. (**b**) Zoomed in voxel (i, j) with its neighbor. The convective heat exchange between (i − 1, j) and (i, j) is calculated using Eq. (15c) and between (i + 1, j) and (i, j) using Eq. (15b). Conduction between voxels (i, j + 1) and (i, j − 1) with (i, j) is calculated using Eq. (15a). The advection between tissue voxels and advection due to the source term are modeled using the second term and third term on RHS of Eq. (15), respectively. (**c**) Multiscale mesh example for heat transfer between a blood vessel and tissue. The arterial element represented using a thick border consists of multiple Arterial Voxels.

### Heat transfer modeling

Each voxel acts as a control volume (CV) exchanging heat with its surroundings. For flow simulation, the CV used is a compartment of the voxel. Thus, for flow simulation, each voxel contains two CVs representing the arterial and venous compartments. The heat transfer simulation incorporates the PBM assumption of instant thermal equilibrium once the blood enters the tissue voxel. Blood enters the tissue voxel in an arterial compartment and perfuses to venous compartment. The arterial compartment, venous compartment and blood in a voxel are at thermal equilibrium, and, therefore, blood perfusion between the arterial and venous compartment in a voxel is ignored.

An illustration of the heat transfer model proposed in this study is shown in Fig. 2. Figure 2a shows a 2D voxel domain to represent an artery, vein, and tissue. For representation purpose, only the terminal elements of arterial and venous tree are shown with the arterial terminal outlet and venous terminal inlet marked with dots. For a given SoI radius, the 2D circles are shown as red for arterial and blue for venous, with their centers at the end of each respective terminal element. Figure 2b shows a zoomed in image of tissue voxel (i, j) with its four neighbors. The neighboring voxels (i, j + 1) and (i, j − 1) are tissues and, voxel (i − 1, j) is part of the arterial terminal element and (i + 1, j) is air.

If a tissue voxel falls within the SoI of any arterial terminal element, it recieves blood from that element. In Fig. 2b, voxel (i, j) is one of the tissue voxels that falls within the SoI, and thus receives a specific amount of blood from the respective arterial element. This source term of blood discussed in the previous section is shown in Fig. 2b for voxel (i, j). Similarly, if the tissue voxel falls within the SoI of a venous terminal element, there is a sink term that collects blood from the respective voxel and transports it to the venous terminal. This sink term is shown for voxel (i, j) as it falls in the SoI of a venous terminal element in Fig. 2. The amount of blood flow related to this source and sink term, depends on the distance of the voxel (i, j) from the arterial and venous terminal element, respectively, as calculated by Eq. (6). If the voxel falls within the SoI of multiple arterial or venous terminal elements, multiple source and sink terms will appear within the voxel.

The blood flow model considers blood perfusion between tissue voxels. This perfusion of blood results in advection. In Fig. 2b the inter-voxel perfusion and the resultant advection between the tissue voxels is shown for (i, j + 1) and (i, j − 1) with (i, j). Blood cannot permeate through a blood vessel wall to a tissue, so there is no direct mass transfer between voxel (i − 1, j) and tissue voxel (i, j). This is one of foundational differences of VoM-PhyS with the VaPor model^28^, where blood perfusion occurs across the vessel walls. Heat is exchanged between a neighboring blood vessel and tissue via convection. Similarly, convective heat exchange takes place with the environment the boundary is exposed to as shown between (i, j) and (i + 1, j). The source term that appears in the tissue voxel brings blood from the respective arterial terminal element as shown in Fig. 2. The mass source term that appears in an arterial compartment of voxel (Eqs. (6), (7)) results in the addition of energy in the voxel due to advection. Each tissue voxel also has metabolic heat generation, represented by the term ${\dot{q}_m}$. Considering these possible heat exchanges across the CV and, the thermal equilibrium of blood and tissue in a voxel, the problem presented is similar to a moving solid with heat generation. An energy equation for this problem is given in Eqs. (1-36), (1-37), and (1-38) in Ref.^42^. The combined form of these equations is shown in Eq. (10).

10 $$ \rho _{{\text{t}}} {\text{c}}_{{{\text{p,t}}}} \frac{{\partial {\text{T}}_{{\text{t}}} }}{{\partial \tau }} = \nabla  \cdot ({\text{K}}_{{\text{t}}} \nabla {\text{T}}_{{\text{t}}} ) - \rho {\text{c}}_{{{\text{p,b}}}} \nabla  \cdot ({\text{T}}_{{\text{t}}} {\tilde{\text{V}}}) + {\dot{\text{Q}}}.$$

In Eq. (10), $\text{T}_\text{t}$ represents the tissue temperature and $\text{V}$ represents the voxel volume. Due to the assumption of thermal equilibrium between blood and tissue in a voxel, the temperature of blood in a voxel is the same as tissue temperature, $\text{T}_\text{t}$. Thermal conductivity and specific heat capacity of tissue are represented by $\text{K}_\text{t}$ and $\text{c}_\text{p,t}$, respectively. The specific heat capacity of blood is represented by $\text{c}_\text{p,b}$ and the velocity of blood across voxels is represented by ${\vec {\text{V}}}$. Traditionally, the velocity vector ${\vec {\text{V}}}$ is assumed to be constant along the flow direction, as there is no mass source or sink term. In the absence of a mass source or sink term, the net sum of mass exchange across the CV will be zero to obey mass conservation. In the current model, there exists mass source and sink terms within a CV which need to be considered in the mass conservation and the resultant energy conservation. In VoM-PhyS framework, blood is considered as the moving solid, and within a tissue voxel, blood and tissue are at same temperature. The coupling of heat equations with flow equations occurs for blood flow resulting in the addition of energy due to blood entering a tissue voxel via source terms. This is incorporated by using Eq. (11), where $\dot{\text{Q}}$ in Eq. (10) is a sum of metabolic heat generation rate ($\dot{q}_m$) and advection. Advection in Eq. (11) is a result of $\text{N}_\text{s}$ source terms that appears in a tissue voxel (arterial compartment). These source terms supply blood directly from the respective $\text{N}_\text{s}$ arteries. The temperature of blood received from these arterial elements by a voxel is given as $\text{T}_\text{i}$.

11 $$\begin{aligned} \dot{\text{Q}} = \dot{q}_m + {\sum _\text{i}^{\text{N}_\text{s}}{\frac{{\text{m}_\text{i}} {\text{c}_\text{p,b}} {\text{T}_\text{i}}}{\text{V}}}}. \end{aligned}$$

The convective heat loss from a voxel to a neighboring blood vessel is given by Eq. (12). ${\text{T}}_\text{t}$ represents the temperature of the tissue voxel next to a blood vessel element $\text{k}$. The blood vessel element can be an artery or vein represented by ${\beta }$. The surface area of the voxel in contact with the blood vessel is shown by $\text{A}_\text{s}$, and the convective heat transfer coefficient between blood and tissue is $\text{h}_\text{b}$. $\text{V}$ represents the voxel volume.

12 $$\begin{aligned} \dot{\text{Q}}_{\beta } = \frac{h_b A_s}{V} (T_{\beta ,k} - T_t), \end{aligned}$$

where

$$\begin{aligned} {\beta } = {\left\{ \begin{array}{ll}\text{a: artery} \\ \text{v: vein} \end{array}\right. }. \end{aligned}$$

Similarly, the convective heat exchange between a voxel and air is given by $\dot{\text{Q}_{\infty }}$ calculated using Eq. (13). Here, $\text{T}_{\infty }$ represents the ambient air temperature and $\text{h}_{\infty }$ represents the convective heat transfer coefficient between air and tissue.

13 $$\begin{aligned} {\dot{\text{Q}}}_{\infty } = \frac{h_{\infty } A_s}{V} (T_{\infty } - T_t). \end{aligned}$$

Combining Eqs. (10), (11), (12) and (13), leads to Eq. (14)

14 $$\begin{aligned} {\rho } \text{c}_\text{p,t}\frac{\partial T_t}{\partial \tau }&= \text{K}_\text{t}\nabla ^2 \text{T}_\text{t} - \rho \text{c}_\text{p,b} \nabla \cdot \tilde{V} \text{T}_\text{t} + {\sum _\text{i}^{\text{N}_\text{s}} \frac{m_i c_{p,b} T_i}{V} + \dot{q}_m} \nonumber \\&\quad + {\frac{h_\infty A_s}{V} (T_{\infty } - T_t) + \frac{h_b A_s}{V} (T_{\beta ,k} - T_t)}, \end{aligned}$$

and

$$\begin{aligned} {\beta } = {\left\{ \begin{array}{ll}\text{a: artery} \\ \text{v: vein} \end{array}\right. }. \end{aligned}$$

Equation (14) is discretized using a first-order Finite Volume Method (FVM) to arrive at Eq. (15).

15 $$ \begin{aligned}   \rho {\text{c}}_{{{\text{p,t}}}} {\text{V}}\frac{{\Delta {\text{T}}_{{{\text{i,j}}}} }}{\tau } =  & \sum\limits_{{{\text{i}} \in {\text{N}}}} {{\text{UA}}\left( {{\text{T}}_{{\beta ,i}}  - {\text{T}}_{{{\text{i,j}}}} } \right)}  + \sum\limits_{{{\text{i}} \in {\text{N}}_{n} }} {{\text{m}}_{{\text{i}}} {\text{c}}_{{{\text{p,b}}}} \left( {{\text{T}}_{{{\text{t,i}}}}  - {\text{T}}_{{{\text{t,j}}}} } 
\right)}  \\     &  + \sum\limits_{{{\text{k}} \in {\text{N}}_{{\text{s}}} }} {{\text{m}}_{{{\text{a,k}}}} {\text{c}}_{{{\text{p,b}}}} \left( {{\text{T}}_{{{\text{a,k}}}}  - {\text{T}}_{{{\text{t,j}}}} } \right) + {\dot{\text{q}}}_{{\text{m}}} } {\text{V,}} \\  \end{aligned}  $$

where

15a $$ {\text{U}} = \left[ {\frac{{{\text{ds}}}}{{2{\text{K}}_{{\text{t}}}}} + \frac{{{\text{ds}}}}{{2{\text{K}}_{{\text{t}}} }}} \right]^{{ - 1}}  = \frac{{{\text{K}}_{{\text{t}}} }}{{{\text{ds}}}}, $$

15b $$ {\text{U}} = \left[ {\frac{{{\text{ds}}}}{{2{\text{K}}_{{\text{t}}} }} + \frac{1}{{{\text{h}}_{\infty } }}} \right]^{{ - 1}} , $$

15c $$ {\text{U}} = \left[ {\frac{{{\text{ds}}}}{{2{\text{K}}_{{\text{t}}} }} + \frac{1}{{{\text{h}}_{{\text{b}}} }}} \right]^{{ -1}} , $$

and

$$\begin{aligned} {\beta } = {\left\{ \begin{array}{ll}\text{a: artery} \\ \text{v: vein} \\ \text{t:tissue} \end{array}\right. }. \end{aligned}$$

Each voxel has six neighbors in 3D and four neighbors in 2D. The voxel neighbors can be any material, so the solution must be robust enough to accommodate the previously-described heat transfer mechanisms. The first term on the right-hand-side in Eq. (15) addresses heat exchange between neighboring voxels. In this term, $\text{N}$ is the total number of neighbors of a voxel. The overall heat transfer coefficient $\text{U}$ varies based on the material of the neighboring voxel. If the neighboring voxel is tissue, then $\text{U} = \text{K}_\text{t}/\text{ds}$ as shown in Eq. (15a). If the neighboring voxel is air or a blood vessel, the value of the overall heat transfer coefficient $\text{U}$ is calculated using Eqs. (15b) or (15c), respectively.

Some neighboring voxels may not supply blood to the voxel under consideration but rather may receive from it due to pressure differential. $\text{N}_\text{n}$ in the second summation term in Eq. (15), represents the number of neighbors from which blood is flowing into the current voxel. $\text{N}_\text{n}$ may be less than or equal to $\text{N}$, depending on the pressure differentials.

The third summation term in Eq. (15) represents the energy delivered to the voxel in via advection from $\text{N}_\text{s}$ number of arterial sources that supply blood to the voxel. The fourth term in Eq. (15) is the heat added to the voxel due to metabolic heat generation.

### Multiscale meshing

The major blood vessels that can be generated from imaging data are modeled as 1D pipe networks and are divided into elements only along the flow direction. The dimensional scale of elemental division may not be the same as the voxel dimension, resulting in different mesh scales. Due to this scale difference, one blood vessel element can traverse multiple tissue voxels along its length. Each blood vessel element acts as a differential cell and the entire element is considered to be at the same temperature. An illustration of this can be seen in Fig. 2a,c. In Fig. 2a, the five voxels tagged as ‘Arterial Voxel’ represent a section of an arterial tree. Figure 2c shows the difference between an arterial element and arterial voxel. The three arterial voxels surrounded by thick black border create one arterial tree element. A similar venous tree element is shown in Fig. 2a. This results, in the blood vessel voxels that fall within the same blood vessel element to be isothermic. In Fig. 2c, voxels (i − 1), (i − 1, j − 1) and (i − 1, j − 2) will have uniform temperature as they fall under the same arterial element. This one element of blood vessel mesh is at a different scale than the tissue voxels, and can be seen in Fig. 2c is surrounded by a total of six tissue voxels. The surrounding tissue voxels are not isothermic and exchanges heat via convection depending on the convective heat transfer coefficient. The energy balance for the blood vessel tree is modeled using Eq. (16).

16 $$\begin{aligned} {\rho _\text{b} c_\text{p,b}} \text{V}_{\beta ,e}\frac{\Delta T_j}{\tau } = \sum _{\beta ,k \in N_{k}} m_k c_{p,b} (T_{\beta ,k} - T_{\beta ,j}) + \sum _{i \in N_i^V} h_b A_i (T_i - T_{\beta , j}). \end{aligned}$$

**Figure 3:**
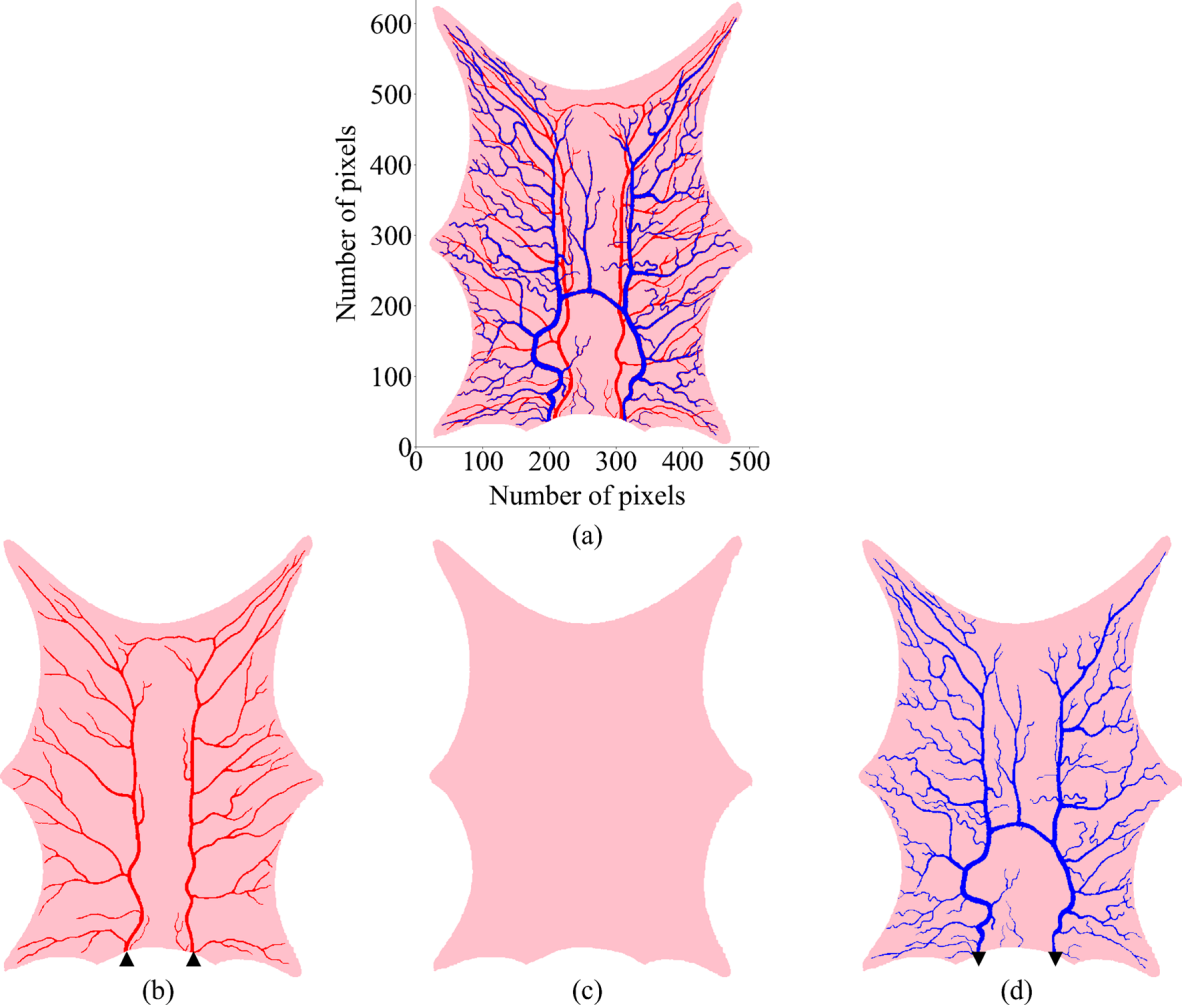
(**a**) Original frog tongue data with arteries in red and veins in blue. The number of pixels in original 2D data are 634 $\times$ 515, with pixel dimensions as 0.063 mm $\times$ 0.064 mm $\times$ 1 mm. The slice was modified for generating three slices each one-third the thickness as follows. (**b**) Layer 1: Arterial tree and tissue. (**c**) Layer 2: Tissue. (**d**) Layer 3: Venous tree and tissue. Filled triangle—Arterial tree root nodes with Dirichlet boundary condition. Filled inverted triangle—Venous tree root nodes with Dirichlet boundary condition.

In Eq. (16), the first term on the RHS considers the advection heat added to the blood vessel element under consideration at temperature $\text{T}_{\beta ,j}$. This advection is the result of the mass flow of blood from one vessel element to another as it flows across the blood vessel network. In the arterial tree, following the direction of flow, the elements divide further, so each element receives blood only from one element. Conversely, in a venous tree, multiple blood vessel elements merge to form single element. Thus, one element may receive blood from multiple elements or from multiple voxels that fall within the SoI. The number of voxels or blood vessel elements that supply blood to the current vessel element is given by $\text{N}_\text{k}$. This blood vessel element is also exchanging heat with surrounding tissue given by the second term. $\text{N}_\text{i}^\text{V}$ shows the tissue voxels that are in immediate contact with the blood vessel element under consideration. These voxels will be at varying temperatures $\text{T}_\text{i}$ and have different surface areas $\text{A}_\text{i}$ in contact with the element.

### Domain modification

The original imaging data of the frog tongue from Ref.^37^ is 2D, as it consists of only a single layer of voxels. Figure 3a shows the original frog tongue data with arterial tree in red and venous tree in blue. The thickness of the slice is 1mm and is available for download from Ref.^37^. A voxel can be artery, vein, or tissue. When such a 2D slice is used for simulation, the source and sink terms generated by the terminals of arteries and veins in the domain are decoupled. This prevents blood perfusion between a tissue-blood interface. As a result, the blood vessels become a separator between source and sink terms and the flow system is discontinuous. To address this, the blood vessels were completely separated from the domain and the entire system was assumed to be tissue^37^. To simulate convective heat exchange at the blood–tissue interface, blood vessel locations are required with reference to surrounding tissue. Hence, the 2D frog tongue domain is converted to 3D by dividing the single slice into three sub-slices across the depth. The top layer contains the main arterial tree, the middle layer consists only of tissue, and the bottom layer contains the venous tree. These layers will be addressed as Layer 1, Layer 2 and Layer 3, respectively, and are shown in Fig. 3.

The order of these layers affects the blood perfusion across the domain. Since the arteries and veins are in the top and bottom layer separated by a tissue, blood entering the domain via source terms in the top layer (Layer 1) must perfuse across the tissue layer (Layer 2) to reach the venous sinks in the bottom layer (Layer 3). If the layers were to be otherwise ordered, the proximity of the source and the sink terms would reduce the perfusion of blood in the tissue layer (Layer 2).

**Table 1:** Parameters used for simulation.

Domain	Parameter	Symbols	Value	Units
Tongue	Specific heat^43^	$\text{c}_\text{pt}$	3421	J kg$^{-1}$ $^\circ$C$^{-1}$
	Density^43^	$\rho _\text{t}$	1090	kg m$^{-3}$
	Thermal conductivity^43^	$\text{K}_\text{t}$	0.49	W m$^{-1}$ $^\circ$C$^{-1}$
	Perfusion^43^	${\alpha }$	1$\times$ 10$^{-6}$	ms kg$^{-1}$
	Arterial permeability^37,39^	${\kappa _\text{a}}$	1 $\times$ 10$^{-12}$	m$^{2}$
	Venous permeability^37,39^	${\kappa _\text{v}}$	5 $\times$ 10$^{-10}$	m$^{2}$
	Metabolic heat gen. rate^44^	$\dot{\text{q}_m}$	0	W m$^{-3}$ s$^{-1}$
Blood	Specific heat^43^	$\text{c}_\text{pb}$	3617	J kg$^{-1}$ $^\circ$C$^{-1}$
	Viscosity^37^	${\mu }$	3$\times$10$^{-3}$	Pa s
	Thermal conductivity^43^	$\text{K}_\text{b}$	0.52	W m$^{-1}$ $^\circ$C$^{-1}$
	Density^43^	${\rho _\text{b}}$	1050	kg m$^{-3}$
	Pressure drop parameter^37^	${\gamma }$	1$\times$10$^{14}$	m$^{3}$
	Arterial inlet pressure^37^	$\text{P}_\text{in}$	10.6	kPa
	Venous outlet pressure^37^	$\text{P}_\text{out}$	1.60	kPa
	Voxel dimensions^37^		64 $\times$ 64 $\times$ 333	$\upmu$m$^{3}$
	Ambient temperature	$\text{T}_{\infty }$	20	C$^{-1}$
	Inlet blood temperature	$\text{T}_{in}$	35	C$^{-1}$

### Simulation parameters

To demonstrate the VoM-PhyS framework, a steady state simulation on the frog tongue shown in Fig. 3 was conducted. Frogs are cold-blooded and have low metabolic heat generation rates^44^. For this study, the metabolic heat generation rate is taken to be zero, and other thermo-physiological parameters are obtained from Refs.^37,39,43^. These parameters are presented in Table 1.

Here, ${\epsilon =}$ 10 mm is used for flow simulation and heat transfer as in Ref.^37^. This value ensures that each voxel has at least one direct source of blood from the arterial tree and at least one sink connecting it to the venous tree. No tissue voxel relies solely on cross-voxel permeability to receive blood from the arterial tree or deliver blood to the venous tree.

**Figure 4:**
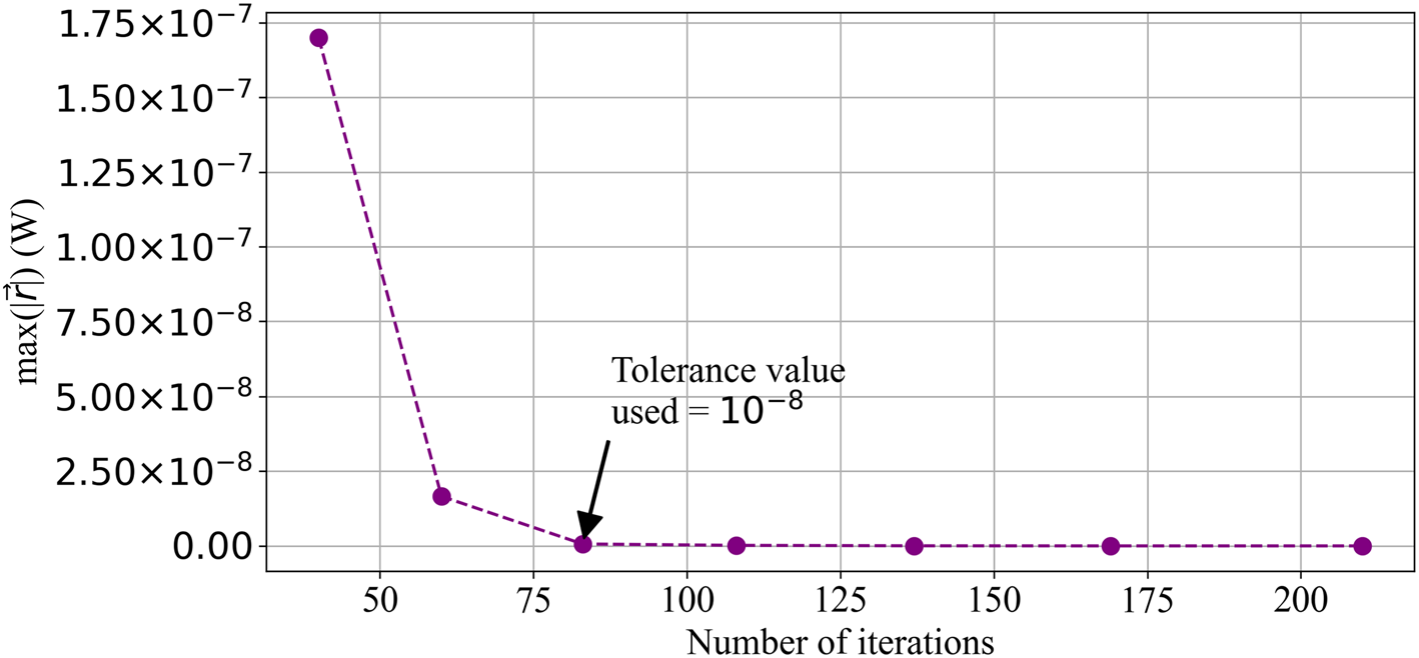
Convergence analysis.

Blood flow matrix ${\mathcal{A}}_{n}$ is generated using Eqs. (1), (7), (8) and (9) and four equations of Dirichlet boundary condition applied at the arterial and venous root nodes. Explicit form of these equations are given in Supplementary Appendix A. An Heat transfer matrix ${\mathcal{A}}_{m}$ is generated using (15), (16) and two equations of Dirichlet boundary condition applied at the arterial root nodes. Explicit equations for heat transfer matrix are given in Supplementary Appendix B. Both matrices ${\mathcal{A}}_{n}$ and ${\mathcal{A}}_{m}$ are sparse with a sparsity of 99.99% and n = 1,111,803 and m = 556,078. These matrices are solved using the Generalized minimal residual (GMRES) method^45,46^. To increase the solver speed, incomplete LU decomposition of the matrices is used as a preconditioner ‘$\text{M}$’. The convergence study conducted for this problem to determine the appropriate tolerance setting is shown in Fig.  4. The residual $\vec {r} = {\mathcal{A}}_m \vec {x} - \vec {b}$ was calculated and the maximum of $|{\vec {r}}|$ is plotted against number of iterations needed in Fig. 4. Maximum of $|{\vec {r}}|$ was selected for convergence analysis as it represents the discrepancy in energy conservation in watts for the obtained solution. At a tolerance value of 1 $\times$ 10$^{-8}$, the maximum residual error is 6.35 $\times$ 10$^{-10}$ W, and continues to reduce further exponentially as the tolerance is decreased. For this study a tolerance of 1 $\times$10$^{-8}$ is used for GMRES function in Python.

### Parameter sensitivity analysis

A numerical sensitivity analysis was conducted to determine how uncertainties in input parameter values propagate through the model and affect the output parameter. The primary aim of this study was to analyze the propagation of error from an input parameter to the final result. A one-at-a-time (OAT) method^37^ was used to calculate normalized sensitivity coefficient ${\mathcal{X}}_{i,w}$ using Eq. (17)

17 $$\begin{aligned} {\mathcal{X}}_{{i,x}} = {\frac{\partial {\theta }_i / {\theta }_i}{\partial x/x}}, \end{aligned}$$

where

17a $$\begin{aligned} \theta _i = \text{T}_i - \text{T}_{\infty }. \end{aligned}$$

The relative sensitivity coefficient ${\mathcal{X}}_{{i,x}}$ is calculated for input variable ‘*x*’ at location ‘*i*’. A temperature offset $\theta$ is used for the sensitivity analysis. The offset is calculated by subtracting a reference Temperature value throughout the entire domain. This makes the coefficient calculated independent of temperature unit. A reference of ambient temperature ($\text{T}_{\infty }$) is used to calculate the temperature offset $\theta _i$ as shown in Eq. (17a). In Eq. (17), subscript ‘i’ represents every voxel and blood vessel element for which temperature is calculated as an output parameter. The relative sensitivity coefficient is represented using an average calculated across the entire domain $\overline{{\mathcal{X}}}_{x}$ using Eq. (18). In Eq. (18), $\mathcal{N}$ is the total number of unknowns for which temperature is calculated. Each input variable is increased by 1% to calculate $\overline{{\mathcal{X}}} _{x}$.

18 $$ \overline{{\mathcal{X}}} _{x}  = \frac{{\sum\nolimits_{{\text{i}}}^{{\mathcal{N}}} {{\mathcal{X}}_{{i,x}} } }}{{\mathcal{N}}}. $$

**Figure 5:**
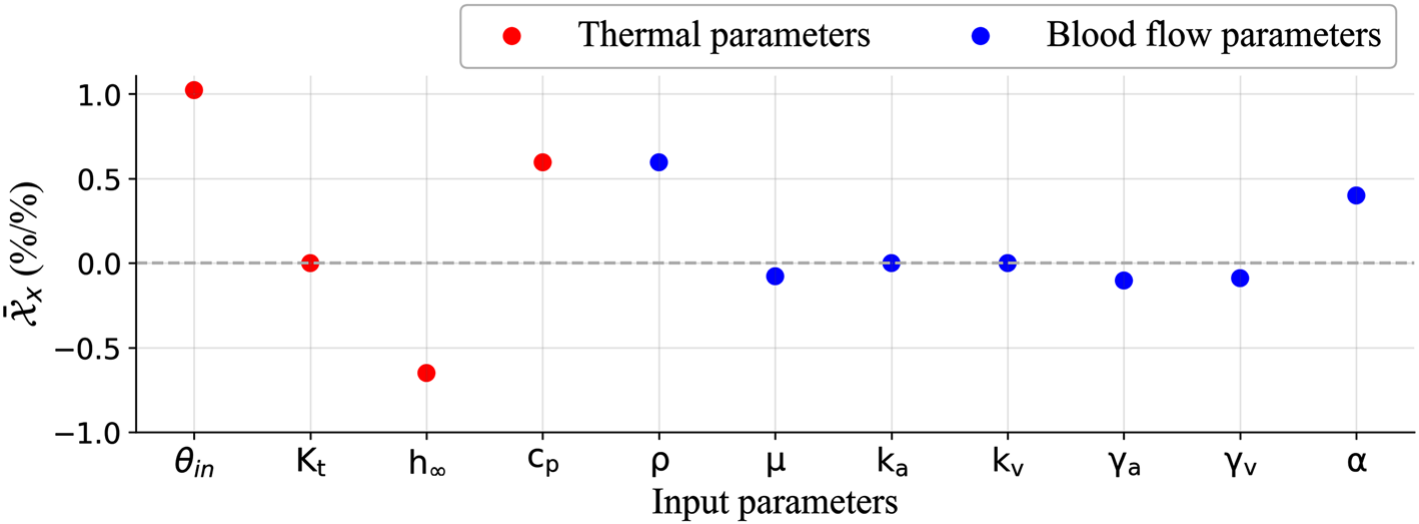
Parameter sensitivity analysis.

In Fig. 5, the relative sensitivity coefficient averaged over the domain ($\bar{\mathcal{X}}_x$) is plotted for various input parameters. $\bar{\mathcal{X}}_x$ represents an average percent change in temperature offset for a 1% change in a given input parameter value. The reference inlet temperature offset $\theta _{in}$ is15 $^\circ$C. When the inlet temperature is specified as 35.15 $^\circ$C to simulate for 1% increase in $\theta _{in}$, an average increase of 1.023% is observed. A 1% increase in inlet temperature resulting in a 1% increase in overall domain temperature is expected. The additional 0.023% observed is due to the tissue voxels closer to ambient temperature. The voxels which are closer to ambient temperature have a smaller $\theta$. A small increase in these voxel temperature results in a higher percentage variation and is seen as the additional 0.023%. Since the metabolic generation is defined to be zero for the simulation, the temperature variation in the domain shows a linear relationship with inlet temperature. Any increase in the inlet temperature corresponds to 1.023% increase in the overall domain temperature. When the convective heat transfer coefficient of ambient air ($\text{h}_{\infty }$) is increased by 1%, the average temperature offset reduces by 0.65%. This is due to more heat being convected out of the domain reducing the overall domain temperature. For a percent increase of arterial to venous compartmental perfusion ($\alpha$), an average of 0.4% increase in temperature was observed. Other flow resistance parameters used in VoM-PhyS ($\text{k}_\text{a}$, $\text{k}_\text{v}$, ${\gamma _\text{a}}$ and $\gamma _{\text{v}}$) did not show an average change of more than ± 0.1% in temperature.

## Results

### Flow simulation

**Figure 6:**
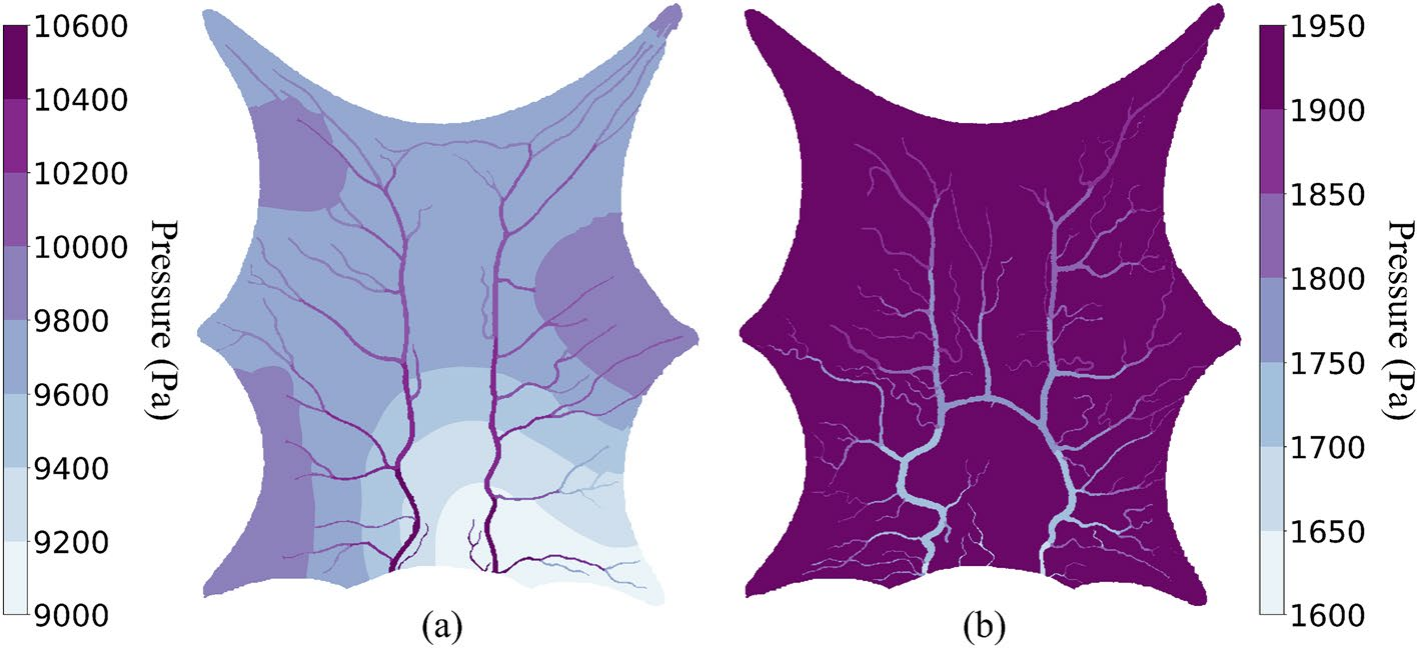
For ${\epsilon = 10 \,\text{mm}}$ (**a**) arterial compartment pressure of Layer 1. (**b**) Venous compartment pressure of Layer 3.

Figure 6 shows the pressure map for flow simulation using a 10 mm SoI radius. Figure 6a, shows the pressure for the arterial compartment of Layer 1. The pressure is highest (10.6 kPa) at the inlet and continues to drop along the blood flow in the arterial tree. The minimum pressure observed in the arterial compartments of the entire domain is around 9 kPa.

Figure 6b shows the pressure map of the venous compartment in Layer 3. The blood in the venous compartment side of the voxel is at approximately 1.95 kPa in the tissue. We observe a pressure drop of around 8 kPa across the capillary bed within the voxels. As the blood flows from the venous compartment of tissue towards the exit through veins, it loses more pressure and the blood exits at 1.6 kPa, which is the given boundary condition.

### PBM assumption: no convective heat exchange between the blood vessel and tissue

**Figure 7:**
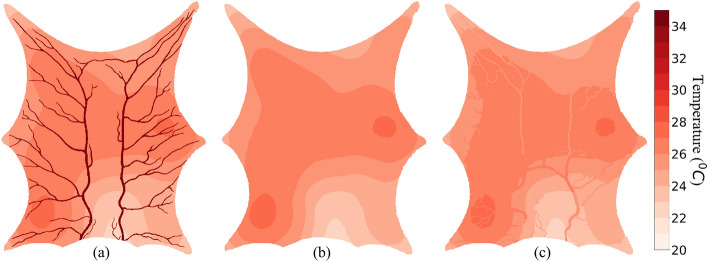
Thermal map using PBM assumption $\text{h}_\text{b} = 0.001$ $\text{W}/{\text{m}^2}$ ${^{\circ }C}$ and ${\epsilon = 10\, \text{mm}}$ (**a**) Layer 1, (**b**) Layer 2, (**c**) Layer 3.

For this simulation, the convective heat transfer coefficient for blood vessels and ambient air were taken to be as 0.001 W m$^{-2}\,{^{\circ }}\hbox {C}^{-1}$ and 20 W m$^{-2}\,{^\circ }\hbox {C}^{-1}$, respectively. The SoI radius was again taken to be 10 mm. The blood temperature leaving the domain was 25.8 $^\circ$C at a steady state. The minimum and maximum tissue temperatures observed were 22.5 $^\circ$C and 27.3 $^\circ$C, respectively. The temperature profiles for the three layers are shown in Fig. 7.

The blood temperature remains constant at 35 $^\circ$C from the entrance to the extremities in the arterial tree and enters tissue at the same temperature. Every voxel that directly receives blood from an artery receives it at 35 $^\circ$C regardless of how far the voxel is from the inlet boundary condition. Once the blood has perfused across the tissue, it enters the venous tree through various sink terms and venous extremities. The temperatures at which blood enters through the extremities in a venous tree are different due to local temperature variations within the tissue domain. Thus, blood enters the venous tree at different temperatures and then experiences no heat exchange with the neighboring voxels.

### WJM assumption: convective heat exchange between blood vessels and tissue

**Figure 8:**
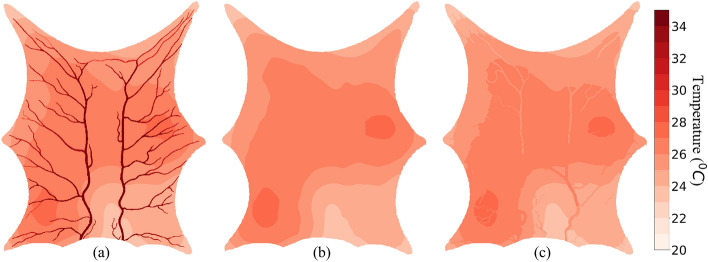
Thermal map using WJM assumption $\text{h}_\text{b} = 10$ $\text{W}/{\text{m}^2}$ ${^{\circ}\text{C}}$ and ${\epsilon = 10\, \text{mm}}$ (**a**) Layer 1, (**b**) Layer 2, (**c**) Layer 3.

For this simulation, the convective heat transfer coefficient for blood vessels and ambient air were taken to be 10 W m$^{-2}\,{^\circ } \hbox {C}^{-1}$ and 20 W m$^{-2}\,{^\circ }\hbox {C}^{-1}$, respectively. The SoI radius was again taken to be 10 mm. The temperature of blood leaving the domain was 25.9 $^\circ$C at a steady state. The minimum and maximum tissue temperatures observed were 23.1 $^\circ$C and 27.4 $^\circ$C. The temperature profiles for the three layers are shown in Fig. 8.

Blood loses heat as it enters the arterial tree and flows towards the extremities to enter the tissue region. Each voxel receives blood at a temperature that depends on its distance from the inlet. The minimum temperature observed in the arterial tree was 27.7 $^\circ$C at the extremity where blood enters the tissue. Similar to results obtained from PBM simulation shown in Fig. 7, blood enters the venous tree at different temperatures but gains heat from the tissue along the flow direction. This effect can be observed via the temperature difference at which the blood leaves the domain through the venous side. Blood leaves at 25.8 $^\circ$C and 25.9 $^\circ$C when the convective heat transfer coefficient between blood and tissue is used as 0.001 W m$^{-2}\,{^\circ }\hbox {C}^{-1}$ and 10 W m$^{-2}\, {^\circ }\hbox {C}^{-1}$, respectively.

## Discussion

**Figure 9:**
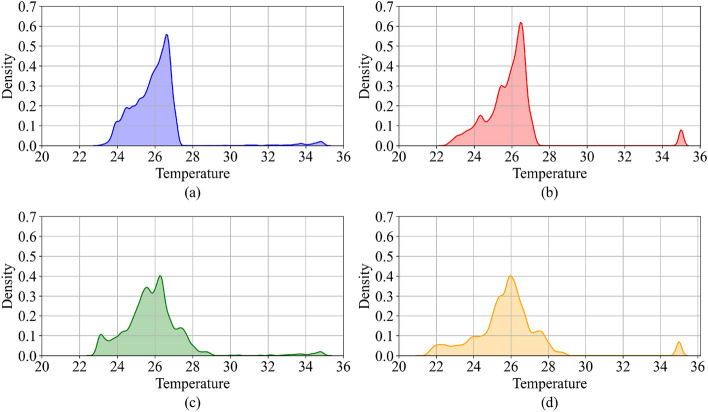
Temperature probability distributions for the frog tongue domain for different simulation parameters. Layer 1, Layer 2, and Layer 3 comprise the 593,769 voxels (blood and tissue) of the domain. Each voxel has a unique temperature. (**a**) ${{\epsilon }} = {10} \,\hbox {mm}$ and ${\text{h}_\text{b} = }$ 10 W m$^{-2}{^\circ } \hbox {C}^{-1}$. (**b**) ${{\epsilon }}$ = 10 mm and $\text{h}_\text{b} =$ 0.001 W m$^{-2}\,{^\circ}\hbox {C}^{-1}$. (**c**) ${{\epsilon =}}$ 5 mm and $\text{h}_\text{b} =$ 10 W m$^{-2}\,{^\circ } \hbox {C}^{-1}$. (**d**) ${{\epsilon =}}$ 5 mm and $\text{h}_\text{b} =$ 0.001 W m$^{-2}\,{^\circ }\hbox {C}^{-1}$.

To study the effect of SoI and convective heat transfer coefficient in VoM-PhyS model, the SoI radius is varied between ${5}\, \hbox {mm}$ and ${10}\, \hbox {mm}$, and heat transfer coefficient between blood vessel and tissue is varied between 0.001 W m$^{-2}\,{^\circ }\hbox {C}^{-1}$ and 10 W m$^{-2}\,{^\circ }\hbox {C}^{-1}$. There are a total of 593,769 voxels in the entire frog tongue domain. Layer 1, Layer 2, and Layer 3 each have 197,923 voxels, which are assigned as either tissue or blood. Each of these voxels has a unique temperature. The temperature distribution for the 593,769 voxels is shown in Fig. 9 for different simulation parameters. These distributions are non-normal skewed distributions, and hence non-parametric statistical tests were considered to study the significance of the differences. To study the effects of SoI radius and convective heat transfer coefficient, the differences in temperature for the voxels comprising the domain were compared; the Wilcoxon signed Rank Test^47,48^ was used to determine significance.

**Figure 10:**
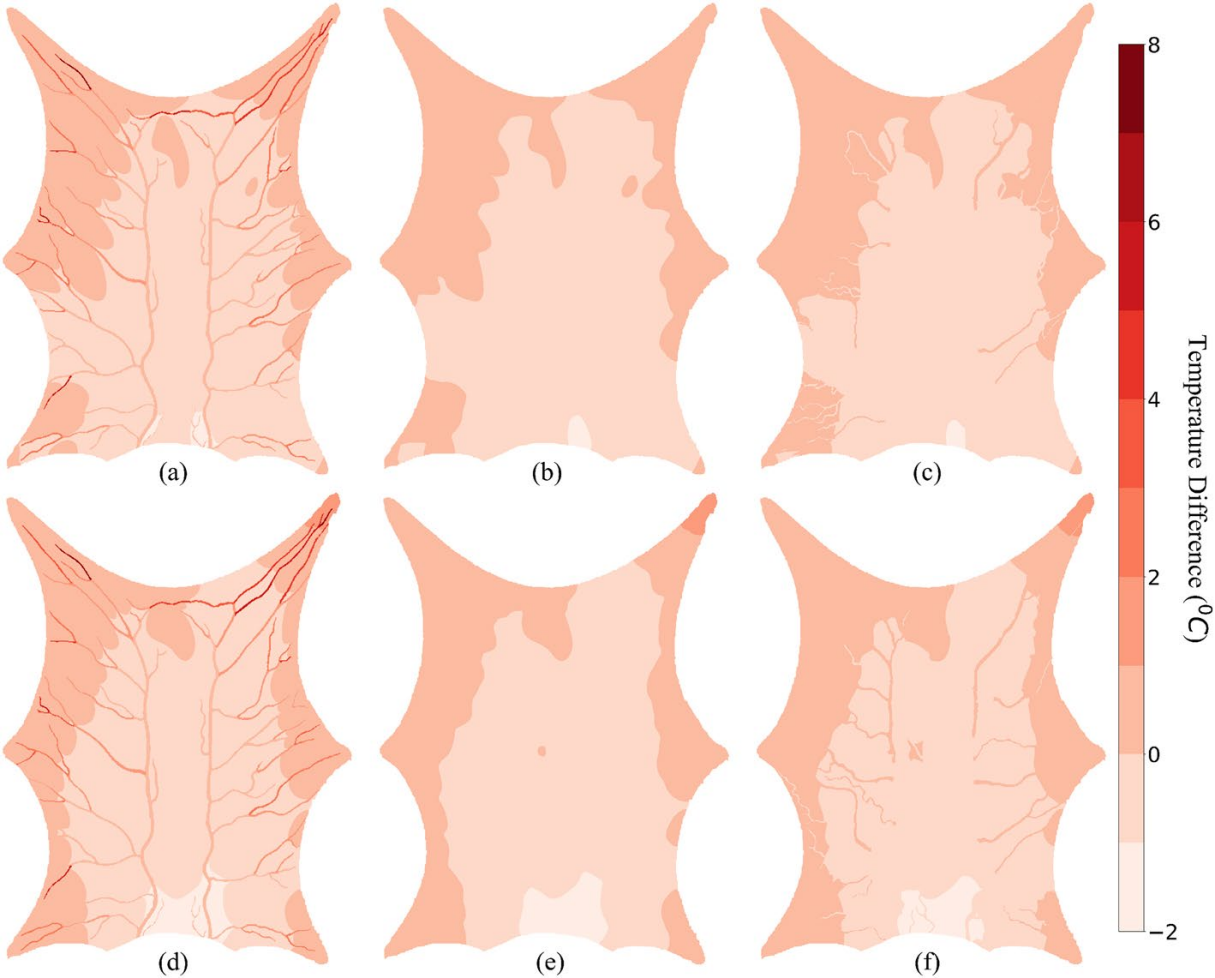
Effect of convective heat exchange between blood and tissue. The positive temperature difference shows the regions which are warmer when we use the PBM assumption and the negative temperature difference shows the region where the WJM assumption results in higher temperature. ${{\epsilon = {10}\,\hbox {mm}}}$: (**a**) Layer 1, (**b**) Layer 2, (**c**) Layer 3 ${{\epsilon = {5}\,\hbox {mm}}}$: (**d**) Layer 1, (**e**) Layer 2, (**f**) Layer 3.

Figure 10 shows the effect of convective heat transfer coefficient between blood vessel and tissue for different SoI radius. A characteristic thermal pattern can be seen in Fig. 10. There is a difference of a maximum of 1 $^\circ$C throughout the tissue domain. The extremities are warmer by 1 $^\circ$C when $\text{h}_\text{b} =$ 0.001 W m$^{-2}\,{^\circ }\hbox {C}^{-1}$ and the region farther away from arterial terminal elements is warmer by 1 $^\circ$C when $\text{h}_\text{b} =$ 10 W m$^{-2}\,{^\circ }\hbox {C}^{-1}$. This difference shows the regions that are dominantly dependent on convective heat exchange between the blood vessel and tissue. The tissue regions farther away from the arterial outlet primarily rely on inter-tissue blood perfusion. When $\text{h}_\text{b} =$ 10 W m$^{-2}\,{^\circ }\hbox {C}^{-1}$ is used, the convective heat exchange warms this region and the extremities receive cooler blood. Blood is at a maximum temperature at the inlet for this domain. Under the PBM assumption ($\text{h}_\text{b} =$ 0.001 W m$^{-2}\,{^\circ }\hbox {C}^{-1}$), blood reaches the entire arterial tree at the same temperature. For the WJM assumption, the blood loses heat along its flow. The highest tissue temperature difference is observed in the region closest to the inlet boundary condition. This is where blood is warmest and, if the convective coefficient is significant, it will exchange heat and raise the temperature of the tissue. This warmer tissue, in return, heats the venous return blood, and thus a warmer blood at the outlet is obtained compared to $\text{h}_\text{b} =$ 0.001 W m$^{-2}\,{^\circ }\hbox {C}^{-1}$ as shown in Table 4. This is the system described by Weinbaum in Ref.^49^. The work of Coccarelli^33^ shows that inner convection plays a crucial role in organ temperatures when there exists a major artery in proximity, and so the results obtained from this simulation support the work of Coccarelli for a micro-scale domain.

**Table 2:** Statistical significance of Fig. 10.

${\epsilon }$ (mm)	Wilcoxon statistic	p-value	$\mathcal{V}_\mathcal{+}$ (%)	$\mathcal{V}_\mathcal{-}$ (%)	$\mathcal{V}_\mathcal{0}$ (%)
5	2.72 $\times 10^{9}$	p < 1 $\times 10^{-3}$	6.13	16.82	77.04
10	5.4 $\times 10^{8}$	p < 1 $\times 10^{-3}$	2.14	9.04	88.8

Parameter $\mathcal{V}_{+}$ represents percentage of domain volume that has ${\Delta \text{T} \ge }$ 1 $^\circ$C.

Parameter $\mathcal{V}_{-}$ represents percentage of domain volume that has ${\Delta \text{T} \le }$ − 1 $^\circ$C. Parameter $\mathcal{V}_0$ represents percentage of domain volume that has − 1 $^\circ$C $< {\Delta \text{T}} <$ 1 $^\circ$C.

The Wilcoxon signed-rank test was performed to study the significance of temperature difference observed in Fig. 10 and the data are shown in Table 2. The test resulted in minimum of sum of the ranks of differences above or below zero as 5.4 $\times 10^{8}$ and 2.7 $\times 10^{9}$ for SoI radius as 5 mm and 10 mm, respectively. For both, the p-value of test was p < 1 $\times 10^{-3}$ which gives the confidence that the results compared are different. For this analysis a minimum of ± 1 $^\circ$C of temperature difference is considered. Parameter $\mathcal{V}$ was calculated which represents the percentage of domain volume that has temperature difference greater than, lower than or equal to zero, and represented using subscripts $+, -$ and 0, respectively. As shown in Table 2, 88.8% of total voxels in the domain do not have a temperature difference of ± 1 $^\circ$C for ${{\epsilon = {10}\, \hbox {mm}}}$. Thus, the effect of convective heat transfer between tissue and voxel is not significantly seen in majority of the domain for a larger ${{\epsilon }}$.

**Figure 11:**
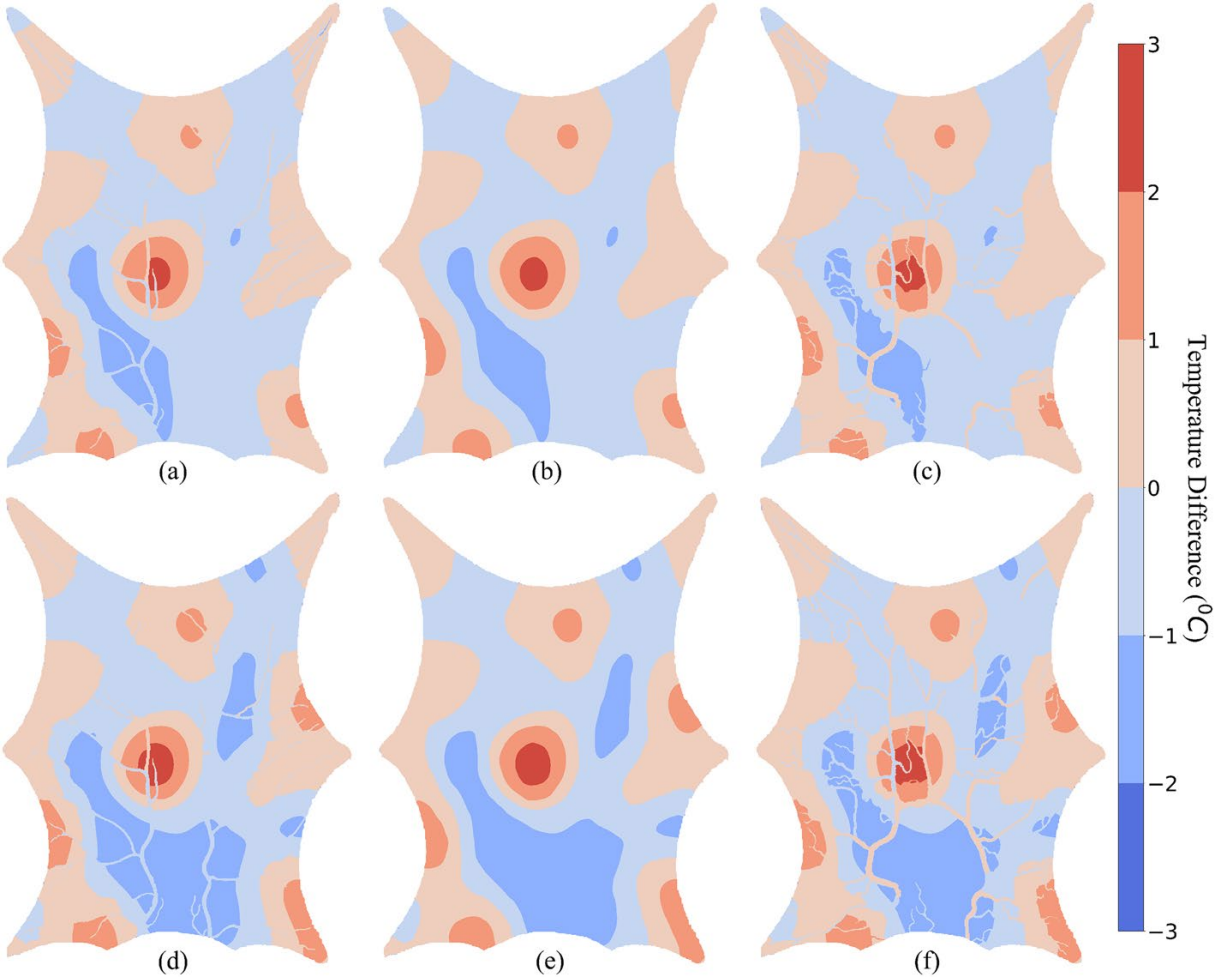
Temperature difference between ${{\epsilon = {5}\, \text{mm}}}$ and ${{\epsilon =}}$ 10 mm. The positive temperature difference shows the region which are warmer when ${\epsilon }$ is smaller. ${\text{h}_\text{b} = }$ 10 W m$^{-2}\,{^\circ }\hbox {C}^{-1}$ : (**a**) Layer 1, (**b**) Layer 2, (**c**) Layer 3 ${\text{h}_\text{b} = }$ 0.001 W m$^{-2}\,{^\circ }\hbox {C}^{-1}$ : (**d**) Layer 1, (**e**) Layer 2, (**f**) Layer 3.

**Table 3:** Statistical significance of Fig. 11.

${\text{h}_\text{b}}$ (W m$^{-2}\,{^\circ }\hbox {C}^{-1}$)	Wilcoxon statistics	p-value	$\mathcal{V}_{+}$ (%)	$\mathcal{V}_{-}$ (%)	$\mathcal{V}_0$ (%)
0.001	25.9 $\times 10^{9}$	p < 1 $\times 10^{-3}$	25.11	32.4	42.48
10	17.3 $\times 10^{9}$	p < 1 $\times 10^{-3}$	19.5	28.33	52.16

Parameter $\mathcal{V}_{+}$ represents percentage of domain volume that has ${\Delta \text{T} \ge }$ 1 $^\circ$C. Parameter $\mathcal{V}_{-}$ represents percentage of domain volume that has ${\Delta \text{T} \le }$ − 1 $^\circ$C. Parameter $\mathcal{V}_0$ represents percentage of domain volume that has − 1 $^\circ$C $< {\Delta \text{T}} <$ 1 $^\circ$C.

Figure 11 shows the effect of varying ${\epsilon }$ when the convective heat transfer coefficient between blood and tissue is constant. The positive temperature difference shows a higher temperature when ${\epsilon }$ is smaller. The SoI controls the volume over which the flow is distributed in the tissue. A larger volume in the SoI results in a lesser of flow to each tissue voxel. Thus, higher temperatures for tissues in close proximity to arterial outlets when ${\epsilon = {5}} \,\hbox {mm}$ than ${\epsilon = {10}\, \hbox {mm}}$ are observed. The negative temperature differences are observed away from arterial outlets. These regions rely on larger SoI to receive blood from arteries and, thus, higher temperatures are observed with ${\epsilon = {10}\, {\hbox {mm}}}$ compared to those observed with ${\epsilon = {5} \,{\hbox {mm}}}$.

Table 3 shows the Wilcoxon signed-rank test results for Fig. 11. Similar to Table 2, a temperature difference of minimum ± 1 $^\circ$C is considered. The minimum of sum of the ranks of differences above or below zero and their respective p-value are given in Table 3. Unlike the results observed in Table 2, more than 50% of total voxels have a temperature difference more than ± 1 $^\circ$C between ${\epsilon }$ = 5 mm and 10 mm for ${\text{h}_\text{b} =}$ 0.001 W m$^{-2}\,{^\circ }\hbox {C}^{-1}$ and only 52.16% of total voxels have a temperature difference less than ± 1 $^\circ$C between ${\epsilon =}$ 5 mm and 10 mm for $\text{h}_\text{b} =$ 10 W m$^{-2}\,{^\circ }\hbox {C}^{-1}$. This shows a significant difference in the thermal maps can be observed based on the SoI radius. Thus the SoI and the corresponding Dirac distribution method used in this work and in Ref.^39^ plays a crucial role in the analysis and accuracy of results.

**Table 4:** Temperature for different simulation conditions.

Parameters	Units	${\epsilon = {10}\,\hbox {mm}}$		${\epsilon = {5}\,{\hbox {mm}}}$		${\epsilon = {2}\,{\hbox {mm}}}$	
Convective coefficient	W m$^{-2}\,{^\circ }\hbox {C}^{-1}$	10	0.001	10	0.001	10	0.001
Inlet temperature	$^\circ$C	35.0	35.0	35.0	35.0	35.0	35.0
Outlet temperature	$^\circ$C	25.9	25.8	25.9	25.8	26.1	26.0
Min arterial temperature	$^\circ$C	27.7	35.0	27.4	35.0	27.4	35.0
Min venous temperature	$^\circ$C	24.1	23.7	23.5	22.9	23.1	22.3
Max venous temperature	$^\circ$C	26.8	26.6	27.6	27.5	29.0	29.1
Min tissue temperature	$^\circ$C	23.1	22.5	22.8	21.5	22.6	21.3
Max tissue temperature	$^\circ$C	27.4	27.3	29.0	28.9	31.6	31.8

The RRT method^32^ used in the VaPor model^28^ generates additional levels of blood vessels that cannot be segmented from medical imaging data. Blowers^28^ demonstrated this on a brain by generating additional blood vessels to simulate hyperthermia. Wang et al.^30^ use the same VaPor model and RRT method for thermal analysis of the skin and foot. In both of these simulations blood perfusion happens across the terminal vessels–tissue interface. This modeled perfusion is uniform across the entire length of the terminal vessel and every voxel that intersects the vessel, receives equal amount of flow. This is very efficient when a single organ is under consideration and the density of the blood vessels can be modelled to the level where such perfusion physically takes place. However, such detailed micro-vasculature at the level of the skin over the entire human is likely to incur a very large computational cost. In the research area of human thermoregulation and human thermal modeling^8,50–52^, accurate localized skin temperature over the entire human body is an important parameter. This skin temperature is regulated by blood flow across the skin and acts as a feedback signal to the hypothalamus for thermal regulation. With advancement in the scientific field thermal manikins are coupled with thermoregulation models^53^. These models require an accurate and local skin temperature and thus a blood vessel network that can provide accurate results. Using the VaPor model and the RRT method to generate blood vessel over the skin on the entire human body will be computationally challenging. If the blood vessels generated using the RRT algorithm do not reach the capillary level, where the assumption of blood perfusion across the vessel wall is valid, it would result in error in pressure drop. VoM-PhyS provides the solution to find the optimum level to generate additional branches using the RRT method and later use SoI technique to supply blood to a volume of region from the terminal vessels.

In the VoM-PhyS framework, the SoI radius ensures that each voxel has at least one source and one sink term. This guarantees that no voxel is left unperfused when the pressure drop across the domain is applied. Many levels of vasculature would be required to achieve this using RRT method, which is tantamount to recreating the entire capillary blood network as obtained from Refs.^1,2,54^. A model that can simulate blood flow and heat transfer needs to map pressure boundary conditions across the vascular network. This becomes more challenging as the relative domain size increases when compared to the size of capillary network. Approximations to these capillaries are required and using porous media methods is one method to represent tissue and capillary bed. The coupling between discretized blood vessels and this porous media domain plays a key role to address the issue of pressure and blood flow. Such a framework can be used to simulate a tracer distribution as shown in Ref.^37^. The VoM-Phys framework provides the ability to model pressure distribution, blood flow and heat transfer irrespective of the domain size relative to the capillary bed.

The VoM-PhyS framework relies on the Dirac distribution method (Eq. (6)) to simulate pre-capillary vessels. Determining the correct value to of the SoI radius ($\epsilon$) remains a challenge. Further research is needed to determine the accurate value of $\epsilon$. The present study considers laminar flow with Newtonian fluid properties throughout the blood vessel network and porous media. The non-Newtonian properties of blood become more pronounced in capillary bed. The dimensions of RBCs are comparable to the diameter of an individual capillary^55,56^ and the non-Newtonian behavior of blood is expected to impact blood circulation and the resultant heat transfer. The effects of non-Newtonian blood flow and transport of individual RBCs are beyond the scope of the present study and future work is needed to better understand resulting influence on thermoregulation. A similar computational model simulating non-Newtonian blood flow in a tumor can be found in Ref.^57^. Zhou et al.^58^ demonstrated the effect of RBCs on the development and modeling of vascular networks. Their findings demonstrate the influence of RBCs on wall shear stress due the particulate nature of RBCs on blood fluid properties. Future research into how to apply these models to larger domains, such as the entire human body, is needed. The domain generated from imaging data relies on the accuracy of the image and the segmentation process involved to create the domain. The diameters of blood vessels obtained during segmentation are dependent on the state of vasodilation or vasoconstriction at the moment when the image was taken. These parameters are expected to affect the blood flow. The future work for the present study includes temporal analysis of blood flow, spatial and temporal variations due to vasomotion, and resultant thermal response. Sensitivity analysis shown in Fig. 5, does not show a significant change for a 1% change in the flow parameters, the local variation of these parameters may be used to simulate local vasomotion and the resultant thermal change to study skin burn^3,4^. Further research on the overall effect of local variation of these parameters is needed. The effect of stair step nature of voxelized mesh was not studied with VoM-PhyS. Possible methods to minimize this error^8^ and a detailed simulation to compare the RRT method with the Dirac distribution function used for coupling 3D and 1D in this work will also be conducted.

## Conclusion

In conclusion, a novel VoM-PhyS framework to model biological domains and simulate a coupled blood-flow and bioheat equation solver is presented. The application of this framework is demonstrated on a frog tongue obtained from literature. The analysis conducted in this paper illustrates the advantages and limitations of the framework due to Dirac distribution method and characteristic radius parameter used for coupling 1D flow with 3D flow.

The value of the characteristic radius plays a critical role in this framework. In-vivo or in-vitro data for different tissue domains may not be available. If a reference experimental value for a spatial and temporal thermal map of the domain is available, the characteristic radius value can be adjusted to match the result. In a transient simulation, a time dependence in the distribution of blood within the SoI is important. A voxel closest to the arterial source point will receive more blood and earlier than a voxel farther away from it. This time dependence is not covered in the present study. It will be addressed and presented in a future work. The challenges can be reduced by obtaining higher resolution images to generate more branches of distinct blood vessels. An increase in the number of distinct blood vessels segmented from the imaging data reduces the error attributed to the virtual branches introduced using characteristic radius.

## Supplementary Information

Supplementary Information.

## Data Availability

The code used for simulation can be found on GitHub at https://github.com/amarerohan/VoM-PhyS. The original frog tongue data can be downloaded from Ref.^37^.
