# Supplementary material for: Modeling a 3-D multiscale blood-flow and heat-transfer framework for realistic vascular systems
Source: Sci Rep. 2022 Aug 26;12:14610. doi: 10.1038/s41598-022-18831-3 (PMC9418225; doi:10.1038/s41598-022-18831-3)
Supplement: Supplementary file 1 — Supplementary Information. [file 41598_2022_18831_MOESM1_ESM.pdf]

Appendix

Appendix A Rearranging the mass balance equations (Eq. (7) and Eq. (8)) results in Eq. (A.1) and Eq. (A.2) for blood flow in arterial and venous compartments of tissue voxels, respectively.

$$\begin{aligned}\left[\sum_{j\in N} t_{ij} + \alpha_i \mathrm{V}_i\right](P_{a,i}) - \sum_{j\in N} t_{ij}(P_{a,j}) - \alpha_i(P_{v,i})\mathrm{V}_i \\ - \sum_{\substack{j\in\mathcal{N}(N_{a,k})\\ k\in N_a^T}} \kappa_{a,jk}\eta^{\epsilon}_{a,k}(x_i - x_k)\mathrm{V}_i(p_{a,j}) \\ - \sum_{\substack{j\in\mathcal{N}(N_{a,k})\\ k\in N_a^T}} \kappa_{a,jk}\eta^{\epsilon}_{a,k}(x_i - x_k)\mathrm{V}_i(p_{a,k}) = 0\end{aligned} \tag{A.1}$$

$$\begin{aligned}\left[\sum_{j\in N} t_{ij} + \alpha_i \mathrm{V}_i\right](P_{v,i}) - \sum_{j\in N} t_{ij}(P_{v,j}) - \alpha_i(P_{a,i})\mathrm{V}_i \\ - \sum_{\substack{j\in\mathcal{N}(N_{v,k})\\ k\in N_v^T}} \kappa_{v,jk}\eta^{\epsilon}_{v,k}(x_i - x_k)\mathrm{V}_i(p_{v,j}) \\ - \sum_{\substack{j\in\mathcal{N}(N_{v,k})\\ k\in N_v^T}} \kappa_{v,jk}\eta^{\epsilon}_{v,k}(x_i - x_k)\mathrm{V}_i(p_{v,k}) = 0\end{aligned} \tag{A.2}$$

Iterating over all the tissue voxel compartments, a sub-matrix of size $(2\mathrm{N_t})$ x $(2\mathrm{N_t} + \mathrm{N_a} + \mathrm{N_v})$ is generated, where $\mathrm{N_t}$, $\mathrm{N_a}$ and $\mathrm{N_v}$ represent number of tissue voxels, number of arterial nodes and number of venous nodes, respectively. The column matrix $b_{2\mathrm{N_t}\times 1}$ is a zero matrix.

Applying the mass conservation equation to every interior node in arterial and venous tree, a set of $\mathrm{N_a^I} + \mathrm{N_v^I}$ equations is generated using Eq. (A.3) and Eq. (A.4). The superscripts T, I and R represent terminal, intermediate and root nodes of blood vessel tree, respectively.

$$\sum_{j\in\mathcal{N}(\mathrm{N_{a,k}})} (\kappa_{a,jk}(p_{a,j} - p_{a,k})) = 0 \tag{A.3}$$

$$\sum_{j\in\mathcal{N}(\mathrm{N_{v,k}})} (\kappa_{v,jk}(p_{a,j} - p_{a,k})) = 0 \tag{A.4}$$

For the number of terminals nodes $(\mathrm{N_a^T}, \mathrm{N_v^T})$, rearranging the pressure continuity Eq. (9), results in Eq. (A.5) and Eq. (A.6) for arterial and venous tree, respectively.

$$\kappa_{a,k}\cdot p_{a,j} - \left[\kappa_{a,k} + \frac{\gamma_a}{\mu}\right]\cdot p_{a,k} + \frac{\gamma_a}{\mu}\sum_{i\in\mathcal{N}(N^v_{a,k})} \eta^{\epsilon}_{a,k}(x_i - x_k)\mathrm{V}_i\cdot P_{a,i} = 0 \tag{A.5}$$

$$\kappa_{v,k}\cdot p_{v,j} - \left[\kappa_{v,k} + \frac{\gamma_v}{\mu}\right]\cdot p_{v,k} + \frac{\gamma_v}{\mu}\sum_{i\in\mathcal{N}(N^v_{v,k})} \eta^{\epsilon}_{v,k}(x_i - x_k)\mathrm{V}_i\cdot P_{v,i} = 0 \tag{A.6}$$

Eq. (A.7) and Eq. (A.8) represent the Dirichlet boundary condition for pressure applied at the root nodes of arterial and venous tree

$$p_{a,k} = \mathrm{P_{in}} \qquad k \in \mathrm{N_a^R} \tag{A.7}$$

$$p_{v,k} = \mathrm{P_{out}} \qquad k \in \mathrm{N_v^R} \tag{A.8}$$

For the matrix equation $\mathcal{A}_\mathrm{n}\mathrm{x} = b$, $\mathrm{n} = 2\mathrm{N_t} + \mathrm{N_a} + \mathrm{N_v}$ and column matrix x is as shown below.

$$\mathrm{x^T} = [\ P_{a,1}\ P_{a,2}\ \dots\ P_{a,N_t}\ P_{v,1}\ P_{v,2}\ \dots\ P_{v,N_t}\ p_{a,1}\ p_{a,2}\ \dots\ p_{a,N_a}\ p_{v,1}\ p_{v,2}\ \dots\ p_{v,N_v}\ ]$$

$\mathrm{x}[1 : \mathrm{N_t}]$ - Pressure in arterial compartment of tissue voxels ($P_{a,i}$)
$\mathrm{x}[\mathrm{N_t} + 1 : 2\mathrm{N_t}]$ - Pressure in venous compartments of tissue voxels ($P_{v,i}$)
$\mathrm{x}[2\mathrm{N_t} + 1 : 2\mathrm{N_t} + \mathrm{N_a}]$ - Pressure in arterial tree nodes ($p_{a,i}$)
$\mathrm{x}[2\mathrm{N_t} + \mathrm{N_a} + 1 : 2\mathrm{N_t} + \mathrm{N_a} + \mathrm{N_v}]$ - Pressure in venous tree nodes ($p_{v,i}$)

The coefficient matrix $\mathcal{A}$ is generated using Eqs. (A.1) to Eqs. (A.8) on respective mesh elements. Column matrix b consists of the pressure boundary condition applied at the arterial and venous nodes.

$$b[k] = \begin{cases} \mathrm{P_{in}} & \text{if } k \in \mathrm{N_a^R} \\ \mathrm{P_{out}} & \text{if } k \in \mathrm{N_v^R} \\ 0 & \text{if } k \notin (\mathrm{N_a^R}, \mathrm{N_v^R}) \end{cases}$$

Appendix B Rearranging Eq. (15) for steady state results in Eq. (B.1). Eq. (B.1) represents energy conservation for a i-th tissue voxel. $\mathcal{N}_i$ consists of voxels that surround tissue voxel 'i' and $\mathcal{N}_n$ represents the neighboring tissue from which blood flows into voxel 'i'. $\mathcal{N}_s$ is a set of arterial outlets which supply blood to voxel 'i'. Eq. (B.1) when iterated over all the tissue voxels in the domain generates a coefficient matrix of size $\mathrm{N_t}$ x $(\mathrm{N_t} + \mathrm{N_a} + \mathrm{N_v})$, where $\mathrm{N_t}$, $\mathrm{N_a}$ and $\mathrm{N_v}$ represent the total number of tissue voxels, arterial elements and venous elements respectively. The column matrix $b_{\mathrm{N_t}}$ is generated using RHS of Eq. (B.1)

$$\begin{aligned}&\sum_{\mathrm{j}\in\mathcal{N}_\mathrm{i}}(\mathrm{UA})_{(\mathrm{ij})}(\mathrm{T}_{\beta,\mathrm{j}}) + \sum_{\mathrm{j}\in\mathcal{N}_\mathrm{n}}\mathrm{m}_{(\mathrm{ij})}\mathrm{c}_{\mathrm{p,b}}(\mathrm{T}_{\mathrm{t,j}}) + \sum_{\mathrm{k}\in\mathcal{N}_\mathrm{s}}\mathrm{m}_{\mathrm{a,k}}\mathrm{c}_{\mathrm{p,b}}(\mathrm{T}_{\mathrm{a,k}})\\ &-\left[\sum_{\mathrm{j}\in\mathcal{N}_\mathrm{i}}(\mathrm{UA})_{(\mathrm{ij})} + \sum_{\mathrm{j}\in\mathcal{N}_\mathrm{n}}\mathrm{m}_{(\mathrm{ij})}\mathrm{c}_{\mathrm{p,b}} + \sum_{\mathrm{k}\in\mathcal{N}_\mathrm{s}}\mathrm{m}_{\mathrm{a,k}}\mathrm{c}_{\mathrm{p,b}}\right](\mathrm{T}_{\mathrm{t,i}}) = -\dot{\mathrm{q}}_\mathrm{m}\mathrm{V}_\mathrm{i}\end{aligned} \tag{B.1}$$

$$\beta = \begin{cases}\mathrm{a : artery}\\ \mathrm{v : vein}\end{cases}$$

Eq. (B.2) and Eq. (B.3) are rearranged form of Eq. (16) and provides the energy conservation for a i-th blood vessel element or arterial and venous tree, respectively. $\mathcal{N}_k$ represent the set of blood vessel elements that supply blood to i-th element and, $\mathcal{N}_v$ is a set of tissue voxels that surround the i-th blood vessel element and exchange heat via convection with it. Eq. (B.2) is iterated over all the blood vessel elements except for the elements that are identified as inlets.

$$\sum_{\mathrm{k}\in\mathcal{N}_\mathrm{k}}\mathrm{m_k c_{p,b}}(\mathrm{T_{a,k}}) + \sum_{\mathrm{j}\in\mathcal{N}_\mathrm{v}}\mathrm{h_b A_j}(\mathrm{T_j}) - \left[\sum_{\mathrm{a,k}\in\mathcal{N}_\mathrm{k}}\mathrm{m_k c_{p,b}} + \sum_{\mathrm{j}\in\mathcal{N}_\mathrm{v}}\mathrm{h_b A_j}\right](\mathrm{T_{a,i}}) = 0 \tag{B.2}$$

$$\sum_{\mathrm{k}\in\mathcal{N}_\mathrm{k}}\mathrm{m_k c_{p,b}}(\mathrm{T_{v,k}}) + \sum_{\mathrm{j}\in\mathcal{N}_\mathrm{v}}\mathrm{h_b A_j}(\mathrm{T_j}) - \left[\sum_{\mathrm{v,k}\in\mathcal{N}_\mathrm{k}}\mathrm{m_k c_{p,b}} + \sum_{\mathrm{j}\in\mathcal{N}_\mathrm{v}}\mathrm{h_b A_j}\right](\mathrm{T_{v,i}}) = 0 \tag{B.3}$$

The last set of equations needed are the boundary conditions. Eq. (B.4) represents the Dirichlet boundary condition of inlet temperature applied to the arterial tree roots.

$$\mathrm{T_{a,k}} = \mathrm{T_{in}} \qquad \mathrm{k} \in \mathrm{N_a^R} \tag{B.4}$$

For the matrix equation $\mathcal{A}_\mathrm{m}\mathrm{x} = b$, $\mathrm{m} = (\mathrm{N_t} + \mathrm{N_a} + \mathrm{N_v})$, and column matrix x is as shown below.

$$\mathrm{x^T} = [\ \mathrm{T_{t,1}}\ \mathrm{T_{t,2}}\ ...\ \mathrm{T_{t,N_t}}\ \mathrm{T_{a,1}}\ \mathrm{T_{a,2}}\ ...\ \mathrm{T_{a,N_a}}\ \mathrm{T_{v,1}}\ \mathrm{T_{v,2}}\ ...\ \mathrm{T_{v,N_v}}\ ]$$

$\mathrm{x^T}[1 : \mathrm{N_t}]$ - Temperature of tissue voxels
$\mathrm{x^T}[\mathrm{N_t} + 1 : \mathrm{N_t} + \mathrm{N_a}]$ - Temperature of arterial tree elements
$\mathrm{x^T}[\mathrm{N_t} + \mathrm{N_a} + 1 : \mathrm{N_t} + \mathrm{N_a} + \mathrm{N_v}]$ - Temperature of venous tree elements

Coefficient matrix $\mathcal{A}$ is generated using Eq. (B.1), (B.2), (B.3) and (B.4) on respective mesh elements.
